# Drought and heat waves associated with climate change affect performance of the potato aphid *Macrosiphum euphorbiae*

**DOI:** 10.1038/s41598-018-37493-8

**Published:** 2019-03-06

**Authors:** Lezel Beetge, Kerstin Krüger

**Affiliations:** Department of Zoology and Entomology, University of Pretoria, Private Bag X20, Pretoria, 0028 South Africa

## Abstract

The combined effect of drought and heat waves on insect-plant interactions is complex and not fully understood. Insects may indirectly benefit from water-deficit stress through increased plant nitrogen levels. Heat stress may have a direct negative effect, yet insect performance may be improved when day-time heat is followed by cooler night temperatures. We show that moderate water-deficit stress (25–30% pot capacity) and high day-night temperatures (30/20 °C) affected *Macrosiphum euphorbiae* on potato (*Solanum tuberosum*) differently than their interactions. Water stress lowered stomatal conductance, and both water and heat stress reduced leaf area. The effect of water stress on nymphal and adult survival depended on temperature. Water stress added to reduced nymphal survival at high but not current (25/15 °C) day-night temperatures. Adult survival at high temperatures was reduced only when combined with water stress. Water stress and high temperatures independently but not interactively reduced the number of daily offspring. Moderate water stress when combined with high temperatures had a negative bottom-up effect on aphid survival even though lower night temperatures aided in the recovery from direct heat stress. Our study illustrates the importance of combining multiple stressors to better understand their impact on insect-plant interactions in the context of climate change.

## Introduction

Understanding the impact of changing climatic conditions on the ecology and evolution of animals and plants has been central to recent research efforts. Global average surface temperature is predicted to increase by 1.5 to 4.5 °C by the end of the 21^st^ century^1^. Due to this increase, major variability in climatic conditions could occur which includes more frequent droughts and higher mid-summer temperatures^1^. These predicted climate changes are likely to affect species distributions, life-history traits, trophic interactions and ecosystem functions^2^. Of particular relevance is how climate change will affect disease and pest outbreaks as this is of direct concern to human health and food production. Several aphid species cause direct feeding damage and transmit plant diseases to crops. They are ideal study organisms for determining the effects of global warming due to their short development times and high reproductive capacity^3^.

Drought and heat waves often occur together, but our understanding of their combined effect on insect herbivores is limited. Whereas numerous studies have been published on the single stressors or combined with other stressors^4,5^, studies on water-deficit stress and high temperature simultaneously are scarce. The interaction between these two stressors and their influence on insect herbivores is complex. Rouault, *et al*.^6^ concluded that aphid outbreaks during and following drought and heat waves in forests in Western and Central Europe varied with aphid species and were difficult to predict. In support of the hypothesis that abiotic stresses improve performance of herbivores^7^, Dale and Frank^8^ observed that urban warming and drought stress combined increased the fitness of a scale insect species on trees^8^.

Environmental factors, such as heat stress, drought and nutrients, influence plant morphology, physiology and chemistry, resulting in bottom-up effects on insect herbivore survival and performance^9–13^. Water-deficit as single stressor may influence aphids indirectly through changes in plant physiology, including an increase in nitrogen levels due to the inhibition of protein metabolism and amino acid synthesis^4,14^. The increase in phloem nitrogen, which consists largely of free amino acids, may benefit the growth and reproduction of phloem-feeding insects such as aphids^15^. However, reduced turgor pressure and water content may render access to increased plant nitrogen more difficult and thus negatively affect phloem-feeding insects on water-stressed plants^4^.

Numerous factors influence the physiology of water-deficit stressed plants and subsequently the performance of insect herbivores. Depending on plant species, cultivar and age, duration and severity of water-deficit stress and aphid species, aphid abundance may be affected positively^16–19^, negatively^16,17,20–22^ or remain unaffected^17–19^.

White^23^ proposed the plant stress hypothesis, initially stating that herbivore performance and population growth are enhanced on stressed plants, especially under water-deficit stress, due to an increase in phloem nitrogen. White^24^ later restricted the plant stress hypothesis to senescence as opposed to flush feeders. On the other hand, the plant vigour hypothesis proposes that herbivores feed preferentially on vigorously growing plants^25^. Such plants may also become stressed, resulting in an increase in nutrients, and flush feeders could benefit from this nutritionally^24^.

Heat waves may influence insects both directly and indirectly through changes in plant traits^26^. Temperature influences aphid population dynamics directly by affecting development, reproduction and survival of aphids^3,27^. High temperature (27.5 to 35 °C) reduces aphid performance, such as reproduction and survival^28–31^. Temperatures fluctuate under natural conditions, especially between day and night^32^. However, few studies have determined the effect of fluctuating temperatures on the biology of aphids. In contrast to constant temperature, fluctuating temperature regimes may improve survivorship and reproduction of aphids at high temperatures^28,30,33^. Davis, *et al*.^30^ observed that *Myzus persicae* (Sulzer) had a higher optimal development threshold and was able to survive extreme temperatures under fluctuating day temperatures, suggesting that aphids were able to recover from heat stress during cooler periods.

The potato aphid, *Macrosiphum euphorbiae* (Thomas) (Hemiptera: Aphididae) originated from North America and has spread throughout the world^34^. This species is known to feed on more than 200 plant species from 20 different families, many of which are herbaceous crops, including potato (*Solanum tuberosum* L., Solanaceae), and ornamental crops^34^. *Macrosiphum euphorbiae* feeds on stems and new flush on the upper part of its herbaceous hosts^35^. It is an economic pest of herbaceous crops by causing direct damages and by transmitting plant viruses such as *Potato virus Y* (PVY) and *Potato leafroll virus* (PLRV) to potato^36^. Severe water-deficit stressed potato plants can reduce adult survival, abundance and biomass of *M. euphorbiae*^21^. The upper lethal temperature for *M. euphorbiae* is 30 °C, at which no nymphs survived to adult stage in the laboratory^33,37^. Furthermore, prolonged high temperatures exceeding 26 °C increased mortality of *M. euphorbiae* in the field^38^.

The study was undertaken because of the paucity of information on the combined effects of drought and heat stress on insect herbivores. We determined the indirect effect of water-deficit stress and heat stress in isolation and when combined, as well as the interaction of the two stressors on aphid biology. We tested the predictions that the performance and population growth of *M. euphorbiae* are enhanced on water-deficit stressed potato plants at fluctuating current temperature but reduced at high fluctuating temperature.

## Results

### Plants

Plants were maintained at current (25/15 °C) and high (30/20 °C) day-night temperatures on either moderately water deficit-stressed (25–30% pot capacity) or well-watered (80–100% pot capacity) potato plants (Table 1). The leaf area was reduced on average by 28% at high temperatures (mean ± SE; 163.82 ± 15.43) compared to plants kept at current temperatures (228.09 ± 18.42; F_1,40_ = 9.265, P = 0.004) and by 33% for water-stressed plants (157.03 ± 12.56) compared to well-watered plants (234.88 ± 19.33; F_1,40_ = 13.592, P = 0.001; n = 11). The interaction between temperature and water level was not significant (F_1,40_ = 0.805, P = 0.375).

**Table 1:** Leaf area (n = 11) and stomatal conductance measured over 42 days (n = 3) of well-watered and moderately water-stressed potato plants maintained at different day-night temperature regimes (mean ± SEM).

Treatment	Leaf area (cm^2^)	Stomatal conductance (mmol m^−2^ s^−1^)
25/15 °C, well watered	276.49 ± 24.93	26.93 ± 0.80
25/15 °C, water stress	179.69 ± 18.30	10.73 ± 0.47
30/20 °C, well watered	193.28 ± 24.54	29.51 ± 1.08
30/20 °C, water stress	134.37 ± 14.99	11.97 ± 0.58

Stomatal conductance (mmol m^−2^ s^−1^) was determined daily as an indirect measure of water stress (Table 1). Over a 42-day period stomatal conductance (mean ± SE) was significantly influenced by water level (F = 99.62, n.d.f, d.d.f. = 1, 5.8, P < 0.001). The stomatal conductance for the two temperatures was significantly lower for water-stressed (11.97 ± 0.38) compared to well-watered plants (28.22 ± 0.68). Neither temperature (F = 0.46, n.d.f, d.d.f. = 1, 5.8, P = 0.523) nor any of the interactions with the exception of day and the day x water interaction influenced stomatal conductance over the 42-day period (day: F = 2.85, n.d.f, d.d.f. = 41, 63.3, P < 0. 0001, day x water: F = 1.58, n.d.f, d.d.f. = 41, 63.3, P = 0.049; n = 42). Leaf nitrogen concentrations did not differ significantly between temperature and water level, nor was the interaction significant (Supplementary Table S1).

### Development and survival of immatures

**Figure 1:**
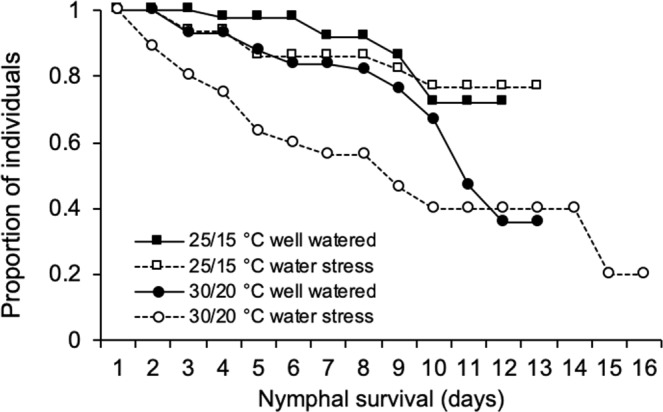
Survival curves for *Macrosiphum euphorbiae* nymphs on well-watered and water-stressed potato plants at current day-night temperatures (25/15 °C) and on well-watered and water-stressed potato plants at high day-night temperatures (30/20 °C). Different letters indicate significant differences between data series (Kaplan–Meier survival regression analysis followed by the log-rank test for pairwise comparisons, P < 0.05; n = 47–71).

Nymphal survival varied with treatment (*Χ*^2^ = 22.945, d.f. = 3, P < 0.001). At current day-night temperatures 72% (n = 47) of nymphs on well-watered and 77% (n = 50) on water-stressed plants developed into adults. At high day-night temperatures 36% (n = 58) of nymphs on well-watered and 20% (n = 71) nymphs on water-stressed plants developed into adults (Fig. 1). On well-watered plants nymphal survival was significantly lower at high temperatures than at current temperatures (*Χ*^2^ = 5.699, d.f. = 1, P = 0.017). Water stress did not influence nymphal survival at current temperatures. At high temperatures nymphal survival was significantly lower on water-stressed plants than on well-watered plants (*Χ*^2^ = 3.957, d.f. = 1, P = 0.047). It was also significantly lower on water-stressed plants at high temperatures compared to well-water plants (*Χ*^2^ = 17.315, d.f. = 1, P < 0.001) and water-stressed plants (*Χ*^2^ = 10.239, d.f. = 1, P < 0.001) at current temperatures. No other pairwise combinations differed significantly.

For nymphs that survived to adulthood instar-specific development time and development time from birth to fourth-instar nymph did not differ between treatments. Interactions between temperature and water regime were not significant except for the development time of the third-instar nymph which was longer on water-stressed plants at high temperatures compared to well-watered and water-stressed plants at current temperatures (F = 5.84, n.d.f, d.d.f. = 1, 153.3, P = 0.017; Table 2).

**Table 2:** Life-history traits of *Macrosiphum euphorbiae* at different day-night temperature and water regimes (means ± SEM).

		25/15 °C Well watered (n = 42)	25/15 °C Water stress (n = 41)	30/20 °C Well watered (n = 39)	30/20 °C Water stress (n = 36)
**A - Duration of development (days)**					
Nymphal stage	Instar 1	2.1 ± 0.1	2.0 ± 0.2	1.8 ± 0.1	1.8 ± 0.1
	Instar 2	1.5 ± 0.1	1.7 ± 0.1	1.6 ± 0.1	1.6 ± 0.1
	Instar 3	2.0 ± 0.1	1.9 ± 0.1	1.8 ± 0.1	2.4 ± 0.2
	Instar 4	2.2 ± 0.1	2.2 ± 0.2	2.5 ± 0.2	2.6 ± 0.2
	Total	7.9 ± 0.3	7.8 ± 0.3	7.7 ± 0.3	8.3 ± 0.3
Adult	Pre-reproductive period	2.5 ± 0.2	2.8 ± 0.2	3.6 ± 0.3	3.6 ± 0.5
	Reproductive period	18.0 ± 1.6	18.0 ± 1.6	16.1 ± 1.5	13.9 ± 1.0
	Post-reproductive period	1.0 ± 0.0	1.0 ± 0.0	1.0 ± 0.0	1.0 ± 0.0
	Longevity	21.3 ± 1.6	21.5 ± 1.5	20.4 ± 1.4	18.3 ± 1.0
**B - Number of offspring produced**					
	Daily reproduction	2.5 ± 0.1	2.2 ± 0.2	1.7 ± 0.1	1.3 ± 0.1
	Total fecundity	47.5 ± 4.5	43.8 ± 4.3	29.6 ± 3.5	21.2 ± 2.6

### Adult longevity and reproductive capacity (fecundity)

**Figure 2:**
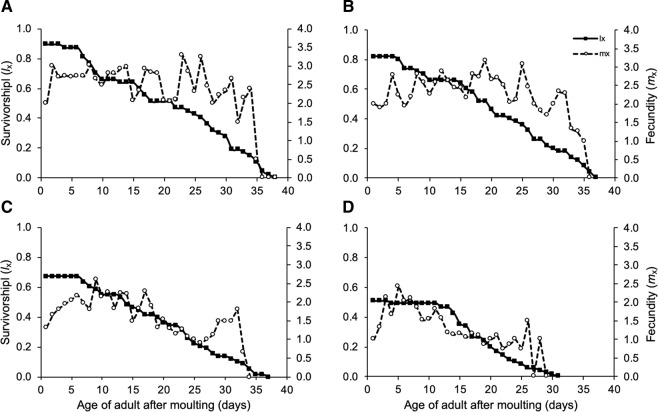
Age-specific survival rate (*l*_x_) and fecundity (*m*_x_) of adult *Macrosiphum euphorbiae* (n = 36–42) on (**A**) well-watered and (**B**) water-stressed potato plants at current day-night temperatures (25/15 °C) and (**C**) on well-watered and (**D**) water-stressed potato plants at high day-night temperatures (30/20 °C).

Temperature and water regime did not influence the duration of the pre-reproductive, the reproductive and the post-reproductive periods or adult longevity, nor were the interactions significant (Table 2). The daily and total number of offspring produced were significantly lower on plants at high day-night temperatures than at current day-night temperatures (daily reproduction: F = 42.42, n.d.f, d.d.f. = 1, 22.8, P < 0.001; total fecundity: F = 9.18, n.d.f, d.d.f. = 1, 5.2, P = 0.028). The total number of offspring produced at high temperatures was on average 43% lower than at current temperatures. Water stress reduced daily (Fig. 2A–D) but not total reproduction (daily reproduction: F = 5.03, n.d.f, d.d.f. = 1, 32.1, P = 0.035). Interactions between temperature and water level for daily and total fecundity were not significant.

### Life table parameters

Temperature and water regime influenced the age-specific survival rate (lx) of adults (*Χ*^2^ = 10.717, d.f. = 3, P = 0.013). Adult survival over time was significantly lower for aphids on water-stressed plants at 30/20 °C than the other treatments (25/15 °C, well-watered: *Χ*^2^ = 8.375, d.f. = 1, P = 0.004; 25/15 °C, water stress: *Χ*^2^ = 8.181, d.f. = 1, P = 0.004; 30/20 °C, well-watered: *Χ*^2^ = 4.473, d.f. = 1, P = 0.034; Fig. 2A–D). No other pairwise comparisons were significant. High temperatures but not water stress influenced growth parameters of *M. euphorbiae* (Table 3). The gross reproductive rate (GRR) and net reproductive rate (R_0_) of *M. euphorbiae* were significantly lower at high temperatures than at current temperatures (GRR: F = 12.22, n.d.f, d.d.f. = 1, 24.5, P = 0.002; R_0_: F = 11.31, n.d.f, d.d.f. = 1, 3.6, P = 0.033). The mean generation time, the intrinsic rate and finite rate of increase, as well as the doubling time, were not affected by temperature or water regime, nor were any interactions significant.

**Table 3:** Life table parameters (mean ± SEM) of *Macrosiphum euphorbiae* at different temperature and water regimes.

Temperature	Water	n	Gross reproductive rate (GRR)	Net reproductive rate (Ro)	Mean generation time (GT)	Intrinsic rate of increase (rm)	Finite rate of increase (λ)	Doubling time (DT)
25/15 °C	Well watered	8	76.45 ± 9.81	48.82 ± 6.23	20.90 ± 1.41	0.17 ± 0.01	1.21 ± 0.01	3.78 ± 0.20
	Water stress	8	62.38 ± 9.50	40.36 ± 8.94	19.86 ± 1.39	0.17 ± 0.01	1.18 ± 0.02	4.47 ± 0.53
30/20 °C	Well watered	7	48.48 ± 8.95	29.18 ± 5.67	19.94 ± 0.82	0.16 ± 0.01	1.18 ± 0.01	4.42 ± 0.32
	Water stress	7	32.37 ± 6.14	18.28 ± 4.49	17.42 ± 0.65	0.15 ± 0.02	1.16 ± 0.02	5.20 ± 0.78

## Discussion

Our study demonstrates that the effects of water and temperature are interlinked. The influence of moderately water-deficit stressed plants on the survival of *M*. *euphorbiae* depended on temperature. Continuous moderate water-deficit stress at high day-night temperatures (30/20 °C) reduced nymphal survival and added to the negative impact of heat stress, whereas water stress had no effect on their survival at current day-night temperatures (25/15 °C). Furthermore, the effect of temperature depended on the water level. Adult survival was reduced only when heat and water stress were combined but not by heat stress alone.

The difference in the response of nymphs and adults to heat and water stress supports the observation that different insect life stages may vary in their response to climate change^39^. In addition, single and combined stressors may affect nymphs and adults differently. Unlike nymphs, where heat and water stress interactively reduced their survival, the reproduction of aphids that survived to adulthood was affected differently by single stressors than by their interaction. Water stress and high temperatures separately reduced the number of daily offspring but there was no interaction between the two stressors.

Aphid reproduction and population growth during heat waves, and even more so when combined with drought, are likely to decrease if the number of nymphs surviving to maturity is reduced. This agrees with a model on the effect of future warming on the abundance of the peach-potato aphid *Myzus persicae*, a vector of PLRV and PVY, in seed potato producing regions in South Africa^40^. The model predicts an increase of 1.9 °C over a 90-year period from 1961 to 2050 in interior potato-producing regions of South Africa resulting in reduced aphid population growth in regions where aphids may reach their thermal limits during the hottest months^40^.

Heat waves together with drought are likely to impact on higher trophic levels by disturbing natural enemy - host interactions and biological control efforts in agriculture. An increase in temperature combined with drought reduced parasitoid emergence from the cabbage aphid, *Brevicoryne brassicae* L.^41^. The reduced survival of nymphs in our study indicate that the decreased survival of parasitoids could be due to lower quality hosts as suggested by Romo and Tylianakis^41^.

Potato plants are considered to be sensitive to water-deficit stress. Different physiological processes, such as transpiration and photosynthesis, can be inhibited or completely stopped because even at low levels of water-deficit stress, potato plants close their stomata^42^. In addition, water-deficit stress leads to a reduction in the growth of potato plants by reducing height and leaf area^42^. Despite reducing the stomatal conductance and leaf area of potato plants in the current study, suggesting that plants were stressed, life history traits of *M. euphorbiae* were not affected, with the exception of a reduction in daily reproduction of aphids that developed on water stressed plants. In contrast to our prediction aphid performance was not enhanced by water stress, possibly because leaf nitrogen concentrations did not increase. The results contradict the plant stress hypothesis^23,24^ that flush feeders would benefit from feeding on stressed plants.

The severity of induced water-deficit stress is important in predicting insect responses^9,43^. Continuous water-deficit stress results in low leaf water content, increases the viscosity of the phloem sap, increases plant defences and potentially leads to reduced feeding of aphids, and limited aphid survival, growth and reproduction^16,21,44,45^. For example, continuous severe drought stress on potato plants for 14 days negatively affected the development time and reproduction of *M. euphorbiae* on *S. tuberosum*^21^. Although severe drought stress may affect aphids negatively, moderate water-deficit stress resulted in higher fecundity and an intrinsic rate of increase in *Brevicoryne brassicae* (L.) and *Myzus persicae* (Sulzer) on *Brassica oleracea* L. (Brassicaceae), as well as higher nitrogen concentrations in plants^19^. However, the influence of water-deficit stress on potato plants may not only depend on the severity but also the timing of the drought stress and the plant species or cultivar being tested^46,47^. In our study, moderate water-water deficit stress did not enhance aphid performance but led to reduced daily but not total reproduction of *M. euphorbiae*. The reduction could be a response to the stress induced increases in ABA and ABA-signalling pathways^45,48^. The results of our study together with the contrasting results of previous work^4,43^ highlight the complexity of direct impact of water stress on plants and subsequent bottom-up effects on insect herbivores.

*Macrosiphum euphorbiae* nymphs were able to survive high day-night temperatures although at a reduced rate. Direct impact of high temperatures can affect aphid mitochondrial processes such as respiration, oxidative phosphorylation, as well as denaturation of proteins and enzymes, resulting in altered functions and cell death^49^. Survival at high temperatures may be attributed to changes in metabolic rates and cellular and physiological processes triggering the production of heat shock proteins, which aid aphids in tolerating temperatures above 27 °C^33^. Furthermore, fluctuating day-night temperatures could also increase survival due to the recovery period from heat stress during cooler conditions at night^30^. In keeping with the findings of Barlow^28^ and Nguyen, *et al*.^33^, no nymphs of *M. euphorbiae* survived to the adult stage in the laboratory at a constant 30 °C (Beetge, unpublished data). However, while aphids may have recovered when exposed to cooler night temperatures during high-temperature periods in the current study, high day-night temperatures still negatively affected nymphal survival and reproduction. Although aphids may be able to adapt to high temperatures to some extent, Hoffmann, *et al*.^50^ suggest that terrestrial ectotherms have a limited ability to change their upper thermal limits.

In addition to reduced nymphal survival, high temperatures negatively affected the reproductive output of *M. euphorbiae*. Similarly, the reproduction of *M. euphorbiae* decreased with an increase in temperature from 25 °C to 30 °C^28,37^ and exposure to heat stress periods reduced the fecundity of *Sitobion avenae* (F.)^51^. This may in part be explained as an indirect effect through the reduced survival of the endosymbiont, *Buchnera aphidicola*, a heat-sensitive bacterium^33^. *Buchnera aphidicola* is located in cells called mycetocytes or bacteriocytes in the abdominal haemocoel of aphids^52^. Aphids survive on the amino acids in the phloem via the synthesis of essential amino acids by *Buchnera*^21,33,52^. A reduction in the abundance of the endosymbiont usually leads to a concomitant reduction in reproductive output in aphids^33^. There was no interactive effect between water level and temperature on total development time, reproductive periods and reproduction of *M. euphorbiae*.

In conclusion, our study provides insights into the impact of moderate water-deficit stress during heat waves on phloem-feeding insects. Moderate water-deficit stress during high day-night temperatures reduced aphid survival but had little effect on aphid population growth parameters of those aphids that reached maturity. Yet, reduced aphid survival and reproduction suggest that *M. euphorbiae* populations could be negatively affected during heat waves, particularly when combined with drought stress, which reduced aphid survival further. Drought and heat waves frequently occur together, and their combined impact on plant - insect herbivore interactions is complex. The study highlights the importance of studying multiple stressors in combination in order to improve our understanding on their effect on plant-insect herbivore interactions in the context of climate change.

## Materials and methods

### Aphid culture

*Macrosiphum euphorbiae* were collected from *Malva parviflora* L. (Malvaceae) at the Experimental Farm of the University of Pretoria (25°45′03.6″S 28°15′28.9″E; Pretoria, Gauteng, South Africa) in June 2009. Viviparous, parthenogenetic *M. euphorbiae* were reared on potato plants (cv. BP1, G0 mini tubers; Rascal Seeds Research Laboratory and Potato Seed Production) and maintained in wooden sleeve cages (55 × 42 × 56 cm) in an environment-controlled room at 25 ± 5 °C, 14 L: 10D photoperiod and current humidity. Light was provided by a combination of Cool White (L30W/640, Osram, Germany) and Fluora (L30W/77, Osram, Germany) fluorescent tubes. The aphid culture was maintained for six months before individuals were used in experiments.

### Plants

Potato plants (cv. BP1, G0 minitubers) were used in the experiments. The potato mini tubers were planted in pots (12.5 cm diameter, 9.5 cm deep) containing 700 ml of sandy soil and coconut husk (4:1 ratio) with 1 ml of dolomitic agricultural lime (calcium 160 g/kg, magnesium 120 g/kg, KKE/CCE (acid) 88%, (resin) 78%; Wonder^TM^ Agro-Serve). Two weeks after planting, *c*. 1.6 g of slow release granular fertilizer (N:P:K 3:1:5, Grovida Khula Kahle^TM^ Fruit and Flower, Durban, South Africa) was added to the soil of each pot and then every 6 weeks after planting. From two weeks after planting micro nutrients (Trelmix trace element solution, 21.3 g Fe/l, 3.0 g Cu/l, 2.7 g Mn/l, 2.3 g Zn/l, 1.0 g B/l, 3.0 g Mo/l and 0.3 g Mg/l; Hubers cc, Howick, South Africa) were sprayed onto the leaves once a week according to the manufacturer’s instructions.

Plants were grown in an environment-controlled room at 25 ± 3 °C, 60–80% relative humidity (RH) and 14 L: 10D photoperiod (Cool white L30W/640 and Fluora L30W/77, Osram, Germany). When plants were 30 days old they were transferred to 200 l growth chambers (Labcon^TM^ 2 LTGC 20, Laboratory Marketing Services cc, South Africa) to precondition them for 30 days to current and elevated day and night temperatures of 25/15 °C and 30/20 °C, respectively, 14 L:10D photoperiod (Cool white L18W/640) and ambient humidity. The current day-night temperature regime was chosen based on mean current summer temperatures in Gauteng, South Africa^53^, which falls into the summer rainfall region with a continental climate.

In addition to the two temperature regimes, two different water regimes were applied to the potato plants during the pre-conditioning phase. Moderate water-deficit stress was induced by maintaining the soil at 25–30% pot capacity. For the well-watered treatment, soil was maintained at 80–100% pot capacity. Increase in the weight of pots due to plant growth was not determined, i.e. plants became more water stressed over time. The initial pot capacity of the soil was calculated by weighing oven-dried soil and water-saturated soil in a pot with an Adam QBW - 15 scale (Adam Equipment Co. Ltd., Milton Keynes, UK). The soil was oven dried at 60 °C for 24 h before weighing. Thereafter it was watered until saturated, and the mass of dry soil subtracted from saturated soil mass to determine the mass of water needed to saturate the soil (100% pot capacity) in the pots. This process was replicated three times for each temperature treatment. To saturate dry soil in the pots (12.5 cm diameter, 9.5 cm), an amount of 180 g of water was required to obtain 100% pot capacity whereas 45 g was needed to maintain plants at 25% pot capacity. Each pot with potato plants was weighed every other day to determine the mass of water needed to obtain either 100% pot capacity (well watered) or 25% pot capacity (water-deficit stress).

Potato plants of approximately the same leaf area at BBCH (Biologische Bundesanstalt, Bundessortenamt und Chemische Industrie) growth stage 19 with nine or more leaves of the main stem unfolded^54^ were chosen for the experiments. Prior to pre-conditioning of plants, leaves from a randomly selected subsample of potato plants were harvested and measured with a LI-3100C Leaf Area Meter (LI-COR, Lincoln, Nebraska, U.S.A.). There was no significant difference in the total leaf area between subsamples of plants assigned to different treatments (analysis of variance (ANOVA), treatment: F_3,44_ = 0.344, P = 0.794, n = 12). The mean leaf area ranged between 151.5 and 162.8 cm^2^. After completion of the experiments, the leaf area was measured again to determine the effect of treatments on plant growth.

The level of water-deficit stress in potato plants was measured indirectly as stomatal conductance (*g*_*s*_), the speed at which water evaporates from the pores in a plant^55^. A plant under water-deficit stress has a low stomatal conductance compared to a well-watered plant^56^. Stomatal conductance of one plant (control plant) for each replicate and treatment was measured with a hand held Decagon Leaf Porometer Model SC-1 (Version 1.31, Decagon Devices, Campbell Scientific Africa, Stellenbosch, South Africa). The sensor head was calibrated to adjust to the experimental environment according to the manufacturer’s instructions every time before taking measurements for a specific treatment. Measurements of abaxial conductance were taken daily for 42 days during the day time light setting from fully expanded terminal leaflets (two top, two middle and two bottom leaves) of each control plant. Measurements were taken from six randomly selected leaflets per plant to account for variation, e.g. exposure to light, leaf age, position, in order to determine a daily mean value for each plant and treatment throughout the experiment. Each treatment was replicated three times.

Leaf nitrogen concentrations were analysed for three plant replicates for each treatment after completion of experiments (Supplementary Table S1).

### Experimental design

Preconditioned 60-day old potato plants were transferred to ventilated glass cages (25 × 25 × 25 cm). Four glass cages containing a single potato plant were placed in each of four growth chambers. In each incubator three of the four potato plants were used to determine the life-history traits of *M. euphorbiae*, and the fourth plant without aphids served to measure stomatal conductance. Each growth chamber simulated different environmental conditions. Two growth chambers each were kept at daily cycles of 25/15 °C day-night temperatures and two growth chambers at daily cycles of 30/20 °C day-night temperatures and 14 L: 10D photoperiod and ambient humidity. The change between day-night temperatures occurred gradually within approximately 30 minutes. Light in each growth chamber was provided by six fluorescent tubes (Cool White L18W/640, Osram, Germany). New tubes were fitted in all growth chambers at the beginning of the experiment in order to standardize light intensity (c. 4880 lux light intensity inside glass cage). At each temperature water-deficit stress (25–30% pot capacity) and well-watered (80–100% pot capacity) regimes were applied to the potato plants in separate incubators. The relative humidity ranged between 35% and 40% for all growth chambers despite efforts to increase relative humidity in the well-watered treatments. The experiment was replicated three times for each temperature and water regime.

A temperature and humidity logger (iButton, Hydrochron Temperature/Humidity Logger, FairBridge Technologies, Sandton, South Africa) was placed in each glass cage within each growth chamber to measure the temperature and humidity at hourly intervals. The mean ( ± SD) temperature in the glass cages was 23.2 ± 2.0 °C during the day and 15.8 ± 2.5 °C at night for the current temperature treatment. The mean temperature for the elevated temperature regime was 27.5 ± 2.7 °C during the day and 20.0 ± 2.3 °C at night.

### Development and survival of immatures

To obtain first-instar nymphs, one adult aptera (wingless) *M. euphorbiae* was confined to one potato leaf in a mesh-covered clip-on leaf cage with an inside diameter of 1.8 cm and height of 1.5 cm. Six leaf cages containing one aphid each, facing the abaxial surface, were attached to six separate leaves on a potato plant for each treatment. Viviparous adult aphids were allowed to deposit first-instar nymphs for 24 h, after which the adult and all but one first-instar nymph were removed from the potato leaf. The single first-instar nymph was confined to the abaxial surface of a potato leaf in a new clip-on leaf cage. Six clip-on leaf cages with one nymph each were used per plant. Individual nymphs were observed once daily for moulting and survival until the last individual from each treatment moulted into the adult stage. The presence of exuviae was used to determine moulting. When calculating development times, nymphs were considered to be 24 h old when the experiment was initiated. Total nymphal development and instar-specific nymphal development duration in days were determined.

### Adult longevity and reproductive capacity

For each treatment, immatures that developed into adults were observed daily for reproduction and survivorship. Newly born offspring were counted and removed daily until the last individual adult in each treatment died. The number of nymphs born to each female was recorded as fecundity and the average daily reproduction was calculated for each aphid.

### Life table parameters

Life table parameters were calculated as described by Birch^57^. Adult age (x), age-specific survival rates (l_x_) and number of offspring produced per female per day (m_x_) were determined for all female aphids used in the study. The life table parameters, gross reproductive rate (GRR = ∑m_x_), net reproductive rate (Ro = ∑l_x_m_x_), mean generation time (GT = ∑ (l_x_m_x_x)/∑ (l_x_m_x_) or GT = ln(Ro)/r), intrinsic rate of increase (r_m_) (∑e-^rx^l_x_m_x_ = 1), finite rate of increase (λ_F_ = e^r^) and doubling time (DT = ln(2)/r) were calculated.

### Data analysis

Data were tested for normality and homogeneity of variances. Two-way analysis of variance (ANOVA) was used to test for differences between leaf area after experiments with temperature and water regime and their interaction as fixed effects. Linear mixed model repeated measurement analysis (REML)^58^ was used to analyse stomatal conductance over 42 days. The fixed effects were specified as day, water, temperature, and all possible interactions, while random effects were specified as trial and trial × treatment and trial × treatment × day interactions. A second-order autoregressive model (AR 2) best modelled the correlation over days. Due to unequal samples sizes (unbalanced design) nymphal development time, adult lifespan, daily reproduction, fecundity and life-table parameters were analysed using linear mixed model analysis (REML) with water, temperature and water × temperature as fixed effects and trial, trial × treatment and trial × treatment × cage as random effects. Where differences between treatments were significant, Fisher’s protected least significant difference (LSD) test was used to separate means. Survival analyses were carried out to test for differences in nymphal survival (survival from newly born nymphs to adults) as well as adult survival between different treatments using the Kaplan-Meier method and the log-rank test for overall and pairwise comparisons. The significance level was set at P < 0.05 for all analyses. Data were analysed with GenStat®^58^ and SPSS (IBM® SPSS® 241 Statistics, version 21).

## Supplementary information

Leaf nitrogen content

## Data Availability

The dataset generated during the current study is available from the corresponding author on reasonable request.

## References

[1] IPCC. Climate change 2013: The physical science basis. Working group I contribution to the fifth assessment report of the intergovernmental panel on climate change. 1–1535 (Cambridge University Press 2013).

[2] Traill LW, Lim MLM, Sodhi NS, Bradshaw CJA (2010). Mechanisms driving change: altered species interactions and ecosystem function through global warming. J. Anim. Ecol..

[3] Harrington, R., Bale, J. S. & Tatchell, G. M. Aphids in a changing climate in *Insects in a Changing Environment*. (eds Harrington, R. & Stork, N. E.) 125–155. (Academic Press 1995).

[4] Huberty AF, Denno RF (2004). Plant water stress and its consequences for herbivorous insects: a new synthesis. Ecology.

[5] Pritchard, J. & Vickers, L. H. Aphids and stress in *Aphids as Crop Pests*. (eds van Emden, H. F. & Harrington, R.) 132–147 (CABI 2017).

[6] Rouault G (2006). Effects of drought and heat on forest insect populations in relation to the 2003 drought in Western Europe. Ann. For. Sci..

[7] Koricheva J, Larsson S, Haukioja E (1998). Insect performance on experimentally stressed woody plants: A Meta-Analysis. Annu. Rev. Entomol..

[8] Dale AG, Frank SD (2017). Warming and drought combine to increase pest insect fitness on urban trees. PLoS ONE.

[9] Mody K, Eichenberger D, Dorn S (2009). Stress magnitude matters: different intensities of pulsed water stress produce non-monotonic resistance responses of host plants to insect herbivores. Ecol. Entomol..

[10] Chen Y, Olson DM, Ruberson JR (2010). Effects of nitrogen fertilization on tritrophic interactions. Arthropod-Plant Interact..

[11] Gutbrodt B, Mody K, Dorn S (2011). Drought changes plant chemistry and causes contrasting responses in lepidopteran herbivores. Oikos.

[12] Han P, Lavoir A-V, Le Bot J, Amiens-Desneux E, Desneux N (2014). Nitrogen and water availability to tomato plants triggers bottom-up effects on the leafminer. Tuta absoluta. Scientific Reports.

[13] Han P (2016). Increased water salinity applied to tomato plants accelerates the development of the leaf miner *Tuta absoluta* through bottom-up effects. Scientific Reports.

[14] Hsiao TC (1973). Plant responses to water stress. Annual Review of Plant Physiology.

[15] Showler, A. T. Water deficit stress - host plant nutrient accumulations and associations with phytophagous arthropods. In *Abiotic Stress - Plant Responses and Applications in Agriculture* (eds Vahdati, K. & Leslie, C.) 387–410 (InTech, 2013).

[16] Hale BK, Bale JS, Pritchard J, Masters GJ, Brown VK (2003). Effects of host plant drought stress on the performance of the bird cherry-oat aphid, *Rhopalosiphum padi* (L.): a mechanistic analysis. Ecol. Entomol..

[17] Rivelli AR, Trotta V, Toma I, Fanti P, Battaglia D (2013). Relation between plant water status and *Macrosiphum euphorbiae* (Hemiptera: Aphididae) population dynamics on three cultivars of tomato. Eur. J. Entomol..

[18] Mewis I, Khan MAM, Glawischnig E, Schreiner M, Ulrichs C (2012). Water stress and aphid feeding differentially influence metabolite composition in *Arabidopsis thaliana* (L.). PLoS ONE.

[19] Tariq M, Wright DJ, Rossiter JT, Staley JT (2012). Aphids in a changing world: testing the plant stress, plant vigour and pulsed stress hypotheses. Agric. For. Entomol..

[20] McVean RIK, Dixon AFG (2001). The effect of plant drought-stress on populations of the pea aphid *Acyrthosiphon pisum*. Ecol. Entomol..

[21] Nguyen TTA, Michaud D, Cloutier C (2007). Proteomic profiling of aphid *Macrosiphum euphorbiae* responses to host-plant-mediated stress induced by defoliation and water deficit. J. Insect Physiol..

[22] Simpson KLS, Jackson GE, Grace J (2012). The response of aphids to plant water stress - the case of *Myzus persicae* and *Brassica oleracea* var. *capitata*. Entomol. Exp. Appl..

[23] White TCR (1969). An index to measure weather-induced stress of trees associated with outbreaks of psyllids in Australia. Ecology.

[24] White TCR (2009). Plant vigour versus plant stress: a false dichotomy. Oikos.

[25] Price PW (1991). The plant vigor hypothesis and herbivore attack. Oikos.

[26] Jamieson MA, Trowbridge AM, Raffa KF, Lindroth RL (2012). Consequences of climate warming and altered precipitation patterns for plant-insect and multitrophic interactions. Plant Physiology.

[27] Bale JS (2002). Herbivory in global climate change research: direct effects of rising temperature on insect herbivores. Global Change Biology.

[28] Barlow CA (1962). Development, survival, and fecundity of the potato aphid, *Macrosiphum euphorbiae* (Thomas), at constant temperatures. Can. Entomol.

[29] Asin L, Pons X (2001). Effect of high temperature on the growth and reproduction of corn aphids (Homoptera: Aphididae) and implications for their population dynamics on the northeastern Iberian Peninsula. Environ. Entomol..

[30] Davis JA, Radcliffe EB, Ragsdale DW (2006). Effects of high and fluctuating temperatures on *Myzus persicae* (Hemiptera: Aphididae). Environ. Entomol..

[31] Zhaozhi, L. *et al*. Differences in the high-temperature tolerance of *Aphis craccivora* (Hemiptera: Aphididae) on cotton and soybean: implications for ecological niche switching among hosts. *Appl. Entomol. Zool*. 9–18 (2017).

[32] Ma C-S, Hau B, Poehling H-M (2004). The effect of heat stress on the survival of the rose grain aphid, *Metopolophium dirhodum* (Hemiptera: Aphididae). Eur. J. Entomol..

[33] Nguyen TTA, Michaud D, Cloutier C (2009). A proteomic analysis of the aphid *Macrosiphum euphorbiae* under heat and radiation stress. Insect Biochem. Mol. Biol..

[34] Millar IM (1990). The aphids (Homoptera: Aphidoidea) of South Africa: an identification guide. Entomology Memoir, Department of Agricultural Development, Republic of South Africa.

[35] Quisenberry, S. S. & Ni, X. Feeding injury in *Aphids as Crop Pests*. (eds van Emden, H. F. & Harrington, R.) 331–352 (CAB International 2007).

[36] Blackman, R. L. & Eastop, V. F. Taxonomic issues in *Aphids as Crop Pests*. (eds van Emden, H. F. & Harrington, R.) 1–29 (CAB International 2007).

[37] Barlow CA (1962). The influence of temperature on the growth of experimental populations of *Myzus persicae* (Sulzer) and *Macrosiphum euphorbiae* (Thomas) (Aphididae). Can. J. Zool..

[38] Walker GP, Nault LR, Simonet DE (1984). Natural mortality factors acting on potato aphid (*Macrosiphum euphorbiae*) populations in processing-tomato fields in Ohio. Environ. Entomol..

[39] Kingsolver JG (2011). Complex life cycles and the responses of insects to climate change. Integrative and Comparative Biology.

[40] van der Waals, J. E., Krüger, K., Franke, A. C., Haverkort, A. J. & Steyn, J. M. Climate change and potato production in contrasting South African agro-ecosystems 3. Effects on relative development rates of selected pathogens and pests. *Potato Res.* **56**, 67–84 (2013).

[41] Romo CM, Tylianakis JM (2013). Elevated Temperature and Drought Interact to Reduce Parasitoid Effectiveness in Suppressing Hosts. PLoS ONE.

[42] van Loon CD (1981). The effect of water stress on potato growth, development, and yield. Am. Potato J..

[43] Sconiers WB, Eubanks MD (2017). Not all droughts are created equal? The effects of stress severity on insect herbivore abundance. Arthropod-Plant Interact..

[44] Isaacs R, Byrne DN, Hendrix DL (1998). Feeding rates and carbohydrate metabolism by *Bemisia tabaci* (Homoptera: Aleyrodidae) on different quality phloem saps. Physiol. Entomol..

[45] Foyer CH, Rasool B, Davey JW, Hancock RD (2016). Cross-tolerance to biotic and abiotic stresses in plants: a focus on resistance to aphid infestation. J. Exp. Bot..

[46] Monneveux P, Ramírez DA, Pino M-T (2013). Drought tolerance in potato (*S. tuberosum* L.), Can we learn from drought tolerance research in cereals?. Plant Science.

[47] Banik P, Zeng W, Tai H, Bizimungu B, Tanino K (2016). Effects of drought acclimation on drought stress resistance in potato (Solanum tuberosum L.) genotypes. Environ. Exp. Bot..

[48] Kerchev PI (2013). Vitamin C and the abscisic acid-insensitive 4 transcription factor are important determinants of aphid resistance in Arabidopsis. Antioxid. Redox Signal..

[49] Kültz D (2005). Molecular and evolutionary basis of the cellular stress response. Annu. Rev. Physiol..

[50] Hoffmann AA, Chown SL, Clusella-Trullas S (2013). Upper thermal limits in terrestrial ectotherms: how constrained are they?. Funct. Ecol..

[51] Jeffs CT (2014). & Leather, S. R. Effects of extreme, fluctuating temperature events on life history traits of the grain aphid. Sitobion avenae. Entomol. Exp. Appl..

[52] Bale, J. S., Ponder, K. L. & Pritchard, J. Coping with Stress in *Aphids as Crop Pests*. (eds van Emden, H. F. & Harrington, R.) 287–303 (CAB International 2007).

[53] Benhin JKA (2006). Climate change and South African agriculture: Impacts and adaptation options. Ceepa Discussion Paper No..

[54] Meier, U. *Growth Stages of Mono- and Dicotyledonous Plants*. 2nd edn. (Federal Biological Research Centre for Agriculture and Forestry 2001).

[55] Collatz GJ, Ball JT, Grivet C, Berry JA (1991). Physiological and environmental regulation of stomatal conductance, photosynthesis and transpiration: a model that includes a laminar boundary layer. Agric. For. Meteorol..

[56] Schapendonk AHCM, Spitters CJT, Groot PJ (1989). Effects of water stress on photosynthesis and chlorophyll fluorescence of five potato cultivars. Potato Res.

[57] Birch LC (1948). The intrinsic rate of natural increase of an insect population. J. Anim. Ecol..

[58] Payne, R. W., Murray, D. A., Harding, S. A., Baird, D. B. & Soutar, D. M. *Introduction to GenStat® for Windows*^*TM*^*(18th Edition)*. (VSN International 2015).

