# Supplementary material for: Drought and heat waves associated with climate change affect performance of the potato aphid Macrosiphum euphorbiae
Source: Sci Rep. 2019 Mar 6;9:3645. doi: 10.1038/s41598-018-37493-8 (PMC6403351; doi:10.1038/s41598-018-37493-8)
Supplement: Supplementary file 1 — Leaf nitrogen content [file 41598_2018_37493_MOESM1_ESM.pdf]

Drought and heat waves associated with climate change affect performance of the potato aphid *Macrosiphum euphorbiae*

Supplementary information

Table

Lezel Beetge & Kerstin Krüger*

Department of Zoology and Entomology, University of Pretoria, Private Bag X20, Pretoria 0028, South Africa

Table S1. Leaf nitrogen content of well-watered and moderately water-stressed potato plants maintained at different day-night temperature regimes (n = 11; mean ± SEM). Nitrogen concentrations were analysed after completion of experiments using the Dumas combustion procedure (AOAC. Official method of analysis 968.06, 2000). The nitrogen concentrations did not differ significantly between temperatures or water levels, nor was the interaction significant (two-way analysis of variance (ANOVA); temperature: $F_{1,40} = 0.020$, $P = 0.888$; water: $F_{1,40} = 0.203$, $P = 0.655$; temperature x water: $F_{1,40} = 0.001$, $P = 0.971$).

Treatment	Nitrogen (g/100 g dry mass)
25/15°C, well watered	4.01 ± 0.63
25/15°C, water stress	4.27 ± 0.46
30/20°C, well watered	4.11 ± 0.47
30/20°C, water stress	4.32 ± 0.54
